# Supplementary material for: A lexical decision task for rapid estimation of crystalized vocabulary knowledge in Thai
Source: PLoS One. 2026 May 4;21(5):e0348126. doi: 10.1371/journal.pone.0348126 (PMC13138683; doi:10.1371/journal.pone.0348126)
Supplement: S1 Fig — (DOCX) [file pone.0348126.s001.docx]

**S1 Figure. Distribution of scores on the 42-item Thai Lexical Decision Task for the 90 participants in Study 2 (interview)**
