# Supplementary material for: A lexical decision task for rapid estimation of crystalized vocabulary knowledge in Thai
Source: PLoS One. 2026 May 4;21(5):e0348126. doi: 10.1371/journal.pone.0348126 (PMC13138683; doi:10.1371/journal.pone.0348126)
Supplement: Attachment — Submitted filename: Response to Reviewers.pdf [file pone.0348126.s003.pdf]

Dear Editor,

Below we provide a response to the comment we received in the decision letter for the previous submission (ID: PONE-D-25-11789).

Editor 1

1. Thank you very much for submitting your manuscript to PLOS ONE, and for responding to our recent requests regarding your submission. After careful evaluation, we have decided that your submission does not meet our publication criteria and must be rejected.

Based on our evaluation of your manuscript and the information you provided, we do not feel that your submission meets our ethical requirements for human subjects research submissions. PLOS ONE requires that research meets all applicable standards for the ethics of experimentation and research integrity (http://journals.plos.org/plosone/s/human-subjects-research). We reserve the right to reject any submission that does not meet our internal ethical standards, which in some cases are more stringent than local ethical standards.

We recognize that you obtained ethical approval from your local ethics committee after you began the study: your ethics approval document is dated March 9, 2023, while participants were recruited beginning in March 3, 2023. For human subjects research, PLOS ONE requires that authors obtain ethics committee approval before beginning the study, and we do not accept retrospective ethical approval.

As a result of these concerns we cannot consider the manuscript for publication at PLOS ONE. I am sorry that we cannot be more positive on this occasion, but hope that you understand the reasons for this decision.

We would like to clarify that the corresponding author had made a typographical mistake in specifying the participant recruitment period in the manuscript. The corresponding author (Suphasiree Chantavarin) had emailed to ask the first author (Graham Pluck), who has access to the participant recruitment information, and Dr. Graham had replied that the recruitment period was between 13/03/2023-08/03/2024. However, when entering this information into the manuscript, the Dr. Suphasiree mistyped 13/03/2023 as 3/03/2023. Below we provide screenshots of this email communication between Dr. Suphasiree and Dr. Graham.

We have corrected this mistake in the revised manuscript (p.8, Line 167).

Since this mistake was due to a typographical error rather than a breach in ethical standards, we humbly request the editor to re-evaluate our submission. We apologize for the inconvenience caused.

Fw: Your Submission CRBEHA-D-24-00112

Thai Le...clean.pdf | PONE-S-25-15325.pdf | Screens...62741.png

You
OK, I'll go ahead and submit it then! ______________________________ อ.ดร.สุภสิรี จันทวรินทร์ Suphasiree Chantavarin, Ph.D. Lecturer and Head of the Cognitive Psychology Area • Faculty of P... Wed 3/5/2025 12:56 PM

Suphasiree Chantavarin
To: g.c.pluck

Reply | Reply all | Forward
Fri 3/7/2025 4:32 PM

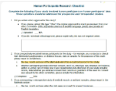

Hi Graham,

The lexical decision manuscript was sent back from PloS ONE because they requested additional information about human subjects data collection (screenshot attached).

They asked us to specify the **day, month, and year** of the **start and end** of the recruitment period for this study (e.g., duration of data collection).

Do you have the info regarding this?

Fw: Your Submission CRBEHA-D-24-00112

Thai Le...clean.pdf | PONE-S-25-15325.pdf | Screens...62741.png

...

Graham Pluck<g.c.pluck@gmail.com>
To: Suphasiree Chantavarin

Reply | Reply all | Forward
Fri 3/7/2025 5:22 PM

13/03/2023 - 08/03/2024

Graham Pluck, B.A., B.Sc., Ph.D., AFBPsS.
Chulalongkorn University,
Faculty of Psychology,
8th Floor,
Borommaratchachonnani Srisattaphat Building,
254 Phayathai Road,
Bangkok,
Thailand 10330

http://www.gpluck.co.uk/
https://www.instagram.com/plucklab/
https://twitter.com/DrGPluck

Fw: Your Submission CRBEHA-D-24-00112

Thai Le...clean.pdf | PONE-S-25-15325.pdf | Screens...62741.png | PONE-D-25-11789.pdf

https://www.instagram.com/plucklab/
https://twitter.com/DrGPluck

...

Suphasiree Chantavarin
To: g.c.pluck

Reply | Reply all | Forward
Mon 3/10/2025 9:44 AM

PONE-D-25-11789.pdf
963 KB

Thanks!

I've resubmitted the manuscript (file attached).

อ.ดร.สุภสิรี จันทวรินทร์
Suphasiree Chantavarin, Ph.D.
Lecturer and Head of the Cognitive Psychology Area
• Faculty of Psychology, Chulalongkorn University
• Bangkok, Thailand
