# Supplementary material for: A lexical decision task for rapid estimation of crystalized vocabulary knowledge in Thai
Source: PLoS One. 2026 May 4;21(5):e0348126. doi: 10.1371/journal.pone.0348126 (PMC13138683; doi:10.1371/journal.pone.0348126)
Supplement: Attachment — Submitted filename: Response to Reviewers (Round 2) v2.pdf [file pone.0348126.s005.pdf]

Dear Editor,

Below we provide a response to the comment we received in the decision letter for the previous submission (ID: PONE-D-25-14461R1) titled "A lexical decision task for rapid estimation of crystalized vocabulary knowledge in Thai".

**Reviewer Comment**	**Action Taken**
**Reviewer 1**	
Most of my previous comments were addressed by the authors. A few additional notes:<br>Pg. 5 - line 104 - typo "let"	We have corrected this typo, highlighted in green on page 5.
Pg. 17 - line 346 - It's confusing to me here. correlation between .1 - .3 are generally considered weak, .3-.5 are considered moderate, and >.5 are considered strong. It's unclear to me why .3 and above are considered "large".	An extra reference has been added to the justify the interpretations used. It is now quite standard in psychology to interpret correlations as 'large' when r > .3. The two citations given both advice that threshold, and are widely cited. The most recent of the guidelines (Gignac & Szodorai, 2016) was cited 784 times last year. The additional citation and reference is indicated in green, on pages 20 and 26.
**Reviewer 3**	
It would be beneficial if the authors could describe the selection criteria as well as the characteristics of the real words included in the list in greater detail. For example, some words belong to royal vocabulary; some have Pali or Sanskrit roots, while others are native Thai words; some are found only in poetry or traditional literature, etc. (As a note, ชลมารค does not mean 'royal barge'; it should be translated as 'watercourse' or 'waterway.') The process of pseudoword formation could also be elaborated. Some pseudowords appear to be modified real words, while others seem to be combinations	Thank you for this helpful suggestion. We have now added a description of the etymology and register of the real words on page 9 (line 196-203) as well as in Table 2 (page 16-19), highlighted in yellow.<br><br>As suggested, we have also made corrections to the English translation of some of the real words (e.g., item # 8, 28, 39), highlighted in pink.<br><br>We also added a more detailed explanation on page 9-10 (line 212-221) on how the pseudowords were created, highlighted in teal.

of parts of real words. Providing this kind of detail would help readers who are not familiar with Thai gain a clearer picture of the nature of the words and pseudowords used in the study.	
On p. 14, the authors state that 20 of the 42 words were relatively high-frequency items, while the remaining words were relatively low-frequency. Did frequency have an effect on participants' performance?	New analysis has been added to address this, the new text is highlighted in grey. This includes statistics presented with the results sections (pp 20-21, 26-27), as well as some interpretation in the General Discussion (page 34).
