# Supplementary material for: A lexical decision task for rapid estimation of crystalized vocabulary knowledge in Thai
Source: PLoS One. 2026 May 4;21(5):e0348126. doi: 10.1371/journal.pone.0348126 (PMC13138683; doi:10.1371/journal.pone.0348126)
Supplement: Attachment — Submitted filename: PONE-D-25-1446.docx [file pone.0348126.s002.docx]

**PLOS ONE**

**A lexical decision task for rapid estimation of crystalized vocabulary knowledge in Thai**

--Manuscript Draft--

| **Manuscript Number:** | PONE-D-25-14461 |
| --- | --- |
| **Article Type:** | Research Article |
| **Full Title:** | A lexical decision task for rapid estimation of crystalized vocabulary knowledge in Thai |
| **Short Title:** | A lexical decision task for rapid estimation of crystalized vocabulary knowledge in Thai |
| **Corresponding Author:** | Suphasiree Chantavarin, Ph.D. Chulalongkorn University Bangkok, THAILAND |
| **Keywords:** | Vocabulary; Mental lexicon; Lexical decision; Premorbid ability; crystalized intelligence; Cognitive assessment; Thai language |
| **Abstract:** | A useful distinction within cognitive and brain sciences is that between fluid and crystallized ability. Although fluid ability is widely studied, crystalized ability, which draws on acquired, declarative semantic knowledge and the mental lexicon, has been less well studied. Partly this is due to the culture and language specificity of assessment methods. We developed and assessed the psychometric properties of a simple 42-item lexical decision task that could be used with Thai speakers to assess the breadth of their crystalized vocabulary knowledge. A large sample of responses from 662 Thai-speaking participants, collected online, was used to refine the scale through exploratory factor analysis, and establish its internai consistency. A smaller sample of 90 participants was interviewed to establish validity of the task as a measure of Thai vocabulary. Large positive correlations were found between the Thai Lexical Decision Task and measures of verbal fluency, particularly in the first 30 seconds of responding, and with other measures of Thai language skill. Temporal stability of the scale was assessed in a subsample of 27 participants. This confirmed that the Thai Lexical Decision Task has little obvious practice effect and excellent test-retest reliability. We also observed the expected positive associations with age, educational level, and self-reported proficiency in the language, supporting the ability of the task to measure acquired, crystalized knowledge. This new task could be used to estimate cognitive ability in Thai adults and shows potential as a measure of premorbid cognitive function for use in neuropsychological assessments. |
| **Order of Authors:** | Graham Pluck, Ph.D. |
|  | Alexis Sirisomboonwong |
|  | Smriti Sitani |
|  | Carl Piaf, M.Sc. |
|  | Suphasiree Chantavarin, Ph.D. |
| **Additional Information:** |  |
| **Question** | **Response** |
| **Financial Disclosure**  Enter a financial disclosure statement that describes the sources of funding for the work included in this submission. Review the submission guidelines for detailed requirements. View published research articles from *PLOS ONE* for specific examples. | The author(s) received no specific funding for this work. |

| This statement is required for submission and **will appear in the published article** if the submission is accepted. Please make sure it is accurate. |  |
| --- | --- |
| **Funded studies**  Enter a statement with the following details:   - Initiais of the authors who received each award - Grant numbers awarded to each author - The full name of each funder - URL of each funder website - Did the sponsors or funders play any role in the study design, data collection and analysis, decision to publish, or preparation of the manuscript? |  |
| Did you receive funding for this work? |  |
| **Competing lnterests**  Use the instructions below to enter a competing interest statement for this submission. On behalf of all authors, disclose any competing interests that could be perceived to bias this  work-acknowledging all financial support and any other relevant financial or non­ financial competing interests.  This statement is required for submission and **will appear in the published article** if the submission is accepted. Please make sure it is accurate and that any funding sources listed in your Funding Information later in the submission form are also declared in your Financial Disclosure statement.  View published research articles from  *PLOS ONE* for specific examples. | The authors declare that no competing interests exist. |

| **NO authors have competing interests**  Enter: *The au/hors have declared that no competing interests exist.*  **Authors with competing interests**  Enter competing interest details beginning with this statement:  *I have read the journal's policy and the au/hors of this manuscript have the following competing interests: [insert competing*  *interests here]* |  |
| --- | --- |
| * typeset |  |
| **Ethics Statement**  Enter an ethics statement for this submission. This statement is required if the study involved:   - Human participants - Human specimens or tissue - Vertebrate animais or cephalopods - Vertebrate embryos or tissues - Field research   Write "N/A" if the submission does not require an ethics statement.  General guidance is provided below. Consult the submission guidelines for detailed instructions. **Make sure that all information entered here is included in the Methods section of the manuscript.** | The procedure, including consent taking, had the approval of local reserach ethics committee (Study Title No. 660010; Approved by the Research Ethics Review Committee at Chulalongkorn University on March 9, 2023) and the research was performed in compliance with all relevant laws and institutional guidelines. |

| **Format for specific study types**  **Human Subject Research (involving human participants and/or tissue)**  Give the name of the institutional review board or ethics committee that approved the study   - lnclude the approval number and/or a statement indicating approval of this research - lndicate the form of consent obtained (written/oral) or the reason that consent was not obtained (e.g. the data were analyzed anonymously)   **Animal Research (involving vertebrate animais, embryos or tissues)**  Provide the name of the lnstitutional Animal Care and Use Committee (IACUC) or other relevant ethics board that reviewed the study protocol, and indicate whether they approved this research or granted a formai waiver of ethical approval   - lnclude an approval number if one was obtained - If the study involved *non-human primates,* add *additional details* about animal welfare and steps taken to ameliorate suffering - If anesthesia, euthanasia, or any kind of animal sacrifice is part of the study, include briefly which substances and/or methods were applied   **Field Research**  lnclude the following details if this study  involves the collection of plant, animal, or other materials from a natural setting:   - Field permit number - Name of the institution or relevant body that granted permission |  |
| --- | --- |
|  |  |
| **Data Availability**  Authors are required to make all data underlying the findings described fully available, without restriction, and from the time of publication. PLOS allows rare exceptions to address legal and ethical concerns. See the PLOS Data Policy and FAQ for detailed information. | Yes - all data are fully available without restriction |

| A Data Availability Statement describing where the data can be found is required at submission. Your answers to this question constitute the Data Availability Statement and **will be published** in **the article,** if accepted. |  |
| --- | --- |
| **Important:** Stating 'data available on request from the author' is not sufficient. If your data are only available upon request, select 'No' for the first question and explain your exceptional  situation in the text box. |  |
| Do the authors confirm that all data underlying the findings described in their manuscript are fully available without restriction? |  |
| **Describe where the data may be found in full sentences. If you are copying our sample text, replace any instances of XXX with the appropriate details.**   - If the data are **held or will be held in a public repository,** include URLs, accession numbers or DOis. If this information will only be available after acceptance, indicate this by ticking the box below. For example: *Ali XXX files are avai/able tram the XXX database (accession number(s)* - If the data are all contained **within the manuscript and/or Supporting Information files,** enter the following: *Ali relevant data are within the manuscript and its Supporting Information files.*    - If neither of these applies but you are able to provide **details of access elsewhere,** with or without limitations, please do so. For example:   *Data cannot be shared public/y because of*  *Data are avai/ab/e tram the XXX lnstitutional Data* Access / *Ethics Committee (contact via*  *for researchers who meet the criteria for* access *to confidential data.*  *The data underlying the results presented in the study are available from (inc/ude the name of the third party* | The Thai Lexical Decision Task is free to use in research without cost, and can be downloaded from: https://gpluck.co.uk/Tests/ |

|    |  |
| --- | --- |
|  |  |

Manuscript	Click here to access/download;Manuscript;Thai Lex Decision	� Task v9_Plos-One.pdf

**1	A lexical decision task for rapid estimation of crystalized vocabulary knowledge in Thaï**

2

3 Graham Pluck, Alexis Sirisomboonwong, Smriti Sitani, Carl Piaf, Suphasiree Chantavarin
4 Faculty of Psychology, Chulalongkom University, Bangkok, Thailand 10330

5

6 **ORCID numbers:**
7 Graham Pluck: 0000-0002-0368-0051
8 Alexis Sirisomboonwong: 0009-0003-2101-8867
9 S1miti Sitani: 0009-0001-8700-5757
10 Carl Piaf: 0009-0004-2260-7775
11 Suphasiree Chantavarin: 0000-0002-5048-9655

**12**

13 **Email addresses:**
14 Graham Pluck: [graham.ch@chula.ac.th](mailto:graham.ch@chula.ac.th)
15 Alexis Sirisomboonwong: [6538085038@student.chula.ac.th](mailto:6538085038@student.chula.ac.th)
16 S1miti Sitani: [6438067638@student.chula.ac.th](mailto:6438067638@student.chula.ac.th)
17 Carl Piaf: [carlpiaf@gmail.com](mailto:carlpiaf@gmail.com)
18 Suphasiree Chantavarin: [Suphasiree.C@chula.ac.th](mailto:Suphasiree.C@chula.ac.th) 19
20 **Corresponding Author:** Suphasiree Chantavarin, Faculty of Psychology, Chulalongkorn
21 University, Bangkok, Thailand 10330. Email: [Suphasiree.C@chula.ac.th](mailto:Suphasiree.C@chula.ac.th)

22

23 **Data Availability:** The raw data supporting these studies is being uploaded to PsychArchives
24 (https://[www.psycharchives.org/).](http://www.psycharchives.org/)) The full web details for the permanent archive will be
25 added at the manuscript revision stage.

# 26 Author contributions:

27 Graham Pluck: Conceptualization, methodology, formal analysis, investigation, Writing

28 original draft.
29 Alexis Sirisomboonwong: Data curation, Investigation, Project administration, Writing

30 review and editing
31 Smriti Sitani: Investigation, Methodology, Writing review and editing
32 Carl Piaf: Formal analysis, Methodology, Writing review and editing
33 Suphasiree Chantavarin: Conceptualization, Investigation, Methodology, Writing review
34 and editing

35

36

37

38

39

40

# 41 Abstract

42 A useful distinction within cognitive and brain sciences is that between fluid and crystallized
43 ability. Although fluid ability is widely studied, crystalized ability, which draws on acquired,
44 declarative semantic knowledge and the mental lexicon, has been less well studied. Partly this
45 is due to the culture and language specificity of assessment methods. We developed and
46 assessed the psychometric properties of a simple 42-item lexical decision task that could be
47 used with Thai speakers to assess the breadth of their crystalized vocabulary knowledge. A
48 large sample of responses from 662 Thai-speaking participants, collected online, was used to
49 refine the scale through exploratory factor analysis, and establish its internal consistency. A
50 smaller sample of 90 participants was interviewed to establish validity of the task as a measure
51 of Thai vocabulary.	were found between the Thai Lexical Decision

Large positive correlations

52 Task and measures of verbal fluency, particularly in the first 30 seconds of responding, and
53 with other measures of Thai language skill. Temporal stability of the scale was assessed in a
54 subsample of 27 participants. This confirmed that the Thai Lexical Decision Task has little
55 obvious practice effect and excellent test-retest reliability. We also observed the expected
56 positive associations with age, educational level, and self-reported proficiency in the language,
57 supporting the ability of the task to measure acquired, crystalized knowledge. This new task
58 could be used to estimate cognitive ability in Thai adults and shows potential as a measure of
59 premorbid cognitive function for use in neuropsychological assessments.

60

# 61 Keywords:

62 Vocabulary, Mental lexicon, Lexical decision, Premorbid ability, Crystalized intelligence,
63 Cognitive assessment, Thai language

# 64 1. Introduction

65 A basic distinction, widely accepted in the various cognitive and neural sciences, is
66

crystallized

-

67 knowledge, particularly related to semantic memory. The most well-known versio
68 distinction between fluid and crystallized intelligence [1], but it is an integral component to
69 many other approaches. One notable example is-component working memory
70 model [2], and another comes from intelligence testing, in which the distinction between
71 [3]. The fluid/crystalized
72 contrast is useful, as the two factors have very different features, particularly in their
73 relationships with formal education [4], developmental trajectories [5], and neurological
74 disorders [6,7]. These are due to the learnt nature of crystalized ability, which increases with
75 age and educational experience, and is quite resilient in the presence of normal and pathological
76 cognitive ageing [5]. Nevertheless, few standardized tests of crystallized ability are available,
77 even in English, as most psychometric studies have focused on fluid abilities such as non-
78 verbal intelligence, working memory, and attention.
79 Fluid ability is characterized by active processing for immediate behavior, such as
80 spatial navigation and decision making; it is very closely associated with complex problem
81 solving [8] and executive functions [9] and may be independent of language skill [10]. In
82 contrast, crystalized ability is closely linked with declarative semantic knowledge, vocabulary,
83 and language skill [11]. However, crystallized ability is more than stored knowledge; it is also
84 linked to intelligence and includes flexible thinking, particularly creativity [12]. It is likely that
85

86 range of cognitive tasks, such as cognitive estimation [13] and verbal fluency [14], but at the
87 neural level, they are dissociable [15].
88 Crystalized ability encompasses much about declarative memory, particularly semantic
89 memory, including various dimensions of general knowledge [16]. Declarative semantic
90 memory also includes the mental lexicon, the set of links between conceptual meaning, and, at
91 its most basic form, the phonemes that indicate those meanings [17]. The different aspects of
92 factual knowledge and the lexicon are very closely associated [11].

# 93 1.1 Methods of assessment of crystalized ability

94

95 method of measuring their crystalized ability. This is because within linguistic cultures, there
96 is a common consensus over the meanings of a finite number of words. By measuring
97 performance on a task of the knowledge of a sample of words, one has an idea of the breadth
98 of the mental lexicon on an individual.
99 One very common method that psychologists use to achieve this is through asking for
100 definitions of words. An example would be a patient/participant being asked what does the
101, and the researcher/clinician scoring the response for accuracy in
102 terms of commonly understood meanings of the word. Such tests are included in many
103 intelligence test batteries, such as the Weschler family of tests [3]. These tests, though, as acts
104 of communication between the patient/participant and researcher/clinician, also likely assess
105 non-lexical processes, such as fluency, mentalizing skill, and social communication adeptness.
106 An alternative is to use lexical decision tasks. In such tasks, real words are presented
107 with pseudowords (i.e., words that appear to be legitimate words in the language but are not
108 actually used in the language). The first such standardized test of this type for psychological
109 research purposes may have been the German -language Mehrfachwahl-Wortschatz-
110 Intelligenztest B (multiple-choice vocabulary intelligence test B; [18]). In that test, German
111 words must be identified from sets composed of one real word plus four pseudowords. In the
112 English language, the Spot-the-Word test [19,20], originally part of the commercially sold

| 113 | Speed and Capacity of Language Processing Test [21], is the most applied lexical decision task |
| --- | --- |
| 114 |  -  |
| 115 |  |
| 116 | udoword. |
| 117 | Lexical decision has several benefits as a testing method. On the part of the |
| 118 | patient/participant, it does not require articulation, and there is an option to guess, thus avoiding |
| 119 | potential embarrassment from being asked to define words one does not understand. On the |
| 120 | part of the researcher/clinician, scoring does not require listening or reading, and so can be |
| 121 | relatively automated, allowing for online or group test administration. It is also objectively |
| 122 | scored, eliminating any variation in scores due to imperfect interrater reliability. |
| 123 | A further benefit is that when lexical knowledge is to be used as a measure of crystalized |
| 124 | ability, it is useful to minimize any fluid processing aspects of task performance. Recognition |
| 125 | of lexical items is likely learnt statistically / implicitly [22] and underlies the processes used in |
| 126 | lexical decision, that is, recognition, not recall. The latter of these requires some level of top- |
| 127 | down executive control, shown by its sensitivity to cognitive load [23], while recognition has |
| 128 | a substantial element of implicit memory, and is generally unaffected by cognitive load [24]. |
| 129 | The reason for this dissociation is that lexical decision tasks can be performed without access |
| 130 | to conceptual knowledge; to respond correctly it is enough to have a feeling that a word is real, |
| 131 | that one has seen that word before, even if one is unable to locate the meaning of the word. |
| 132 | **1.2 Availability of validated lexical decision tasks** |
| 133 | Few standardized lexical decision tasks are available for clinical or research use. One |
| 134 | reason for this is that they are, by their nature, language and culture specific. The Spot-the- |
| 135 | Word Test was developed in the UK and uses British English words, indeed some words (e.g., |
| 136 |  are not listed in dictionaries from the USA, |
| 137 | casting some doubt on its validity within that country. And of course, such tests are not valid |

138 for people who speak languages other than English. Consequently, localized versions need to
139 be developed.
140 Lexical decision tasks very similar to the Spot-the-Word Test have been developed for
141 use in several languages, including French [25], Spanish [26], and Brazilian Portuguese [27].
142 There is also a version in Swedish that uses a different format but is still a lexical-decision task
143 [28].

# 144 1.3 Reason for the current study

145 No standardized test of crystalized ability exists for clinical or psychological research
146 use in the Thai language. Development of such a task would assist future research in
147 psychology and the behavioral sciences within the country. Here, we describe studies of the
148 validity and reliability of a Thai Lexical Decision Task. Study 1 reports the development of the
149 task and the use of factor analysis with a large online sample to refine the scale and to establish
150 its internal consistency. Study 2 aimed to establish the validity of the task by investigating
151 performance correlations between the lexical decision task and measures of Thai verbal
152 fluency. Finally, Study 3 aimed to assess the test-retest reliability of the scale in a subsample

153

154

of participants.

# 155 2. Study 1: Task development and factor analysis with an online sample

156 **2.1. Method**

## 157 2.1.1. Aims, study design and hypotheses

158 The aim of this study was to develop an initial set of words and pseudowords to explore
159 item difficulty and scale unidimensionality with a large sample of participants. An online study
160 was conducted with an initial version of the Thai Lexical Decision Task. We also hypothesized
161 that as a measure of acquired crystalized knowledge, scores on the task would be positively
162 associated with age, education level, and self-rated language proficiency.

## 163 2.1.2. Participants

164 A total of 994 participants were recruited online from the Chulalongkorn University
165 community. The inclusion criteria to participate were that an individual was aged 18 or above,
166 a Thai national, and was a fluent native Thai speaker (exposed to Thai language before the age
167 of 5). The recruitment period was between March 13, 2023 to March 8, 2024.
168 The mean age of the whole sample was 32.6 years (*SD* = 7.1, range = 18-79), and most
169 identified as female, 641/994 (65%). However, many cases were excluded during data quality
170 processing, the actual analyzed sample is thus described in Section 2.2.2.

## 171 2.1.3. Development of the initial version of the Thai Lexical Decision Task

172 To develop a large set of target words for the Thai Lexical Decision Task, we initially
173 selected 100 real Thai words. All words were selected to be concrete concepts that were
174 imageable; the relevance of this feature is described later in Section 5.0. Since participant
175 performance is evaluated based on their accuracy rather than their response times, the task had
176 to be designed to be moderately difficult. Thus, we purposely selected some words that had
177 low frequency of use (e.g.,,,) so that these words will not be easily distinguishable
178 from nonwords. We also included words that were relatively common and familiar (e.g.,,
179,). The frequency of the selected words within the Thai language varied from < 1 to
180 70.2 per million (*M* = 5.9, *SD* = 13.7), estimated using the Thai web 2018 (thTenTen18) corpus
181 from the Sketch Engine website (https://[www.sketchengine.eu/).](http://www.sketchengine.eu/)) The word length ranged from
182 2-7 characters (*M* = 4.1, *SD* = 1.2), defined as the number of consonant and vowel characters
183 excluding the vowels and tone marks located above or below those characters. The number of
184 syllables ranged from 1-4 (*M* = 2.2, *SD* = 0.7). Research assistants looked up each word in
185 search engines and dictionaries to verify that each of the words had meaning in the Thai
186 language.
187 A list of 100 pseudowords was created to be paired with the real words. The
188 pseudowords were designed to all be pronounceable and to closely resemble real words. The
189 length of the pseudowords ranged from 2-8 characters (*M* = 4.2, *SD* = 1.2), and the approximate
190 number of syllables ranged from 1-4 (*M* = 2.0, *SD* = 0.8). The pseudowords contained a similar
191 set of letters as the real words, containing high-frequency letters (e.g.,,,) as well as lower-
192 frequency letters (e.g.,,,), combined in different ways. Research assistants verified through
193 online searches that the pseudowords did not have a conventional meaning in Thai language.
194 Real words and pseudowords were randomly paired to create the 100 test items. Since the
195 random pairing resulted in some very easy items (e.g., a high-frequency real word was paired
196 with a pseudoword that contained low-frequency letters, thus providing a cue that it was a
197 pseudoword), according to pilot data, we manually switched some pairings to avoid creating
198 real word - pseudoword pairs that were too easy.

## 199 2.1.4. Procedure

200 The procedure, including written consent taking, had approval of the local research
201 ethics committee (Study Title No. 660010; Approved by the Research Ethics Review
202 Committee at Chulalongkorn University on March 9, 2023) and the research was performed in
203 compliance with all relevant laws and institutional guidelines. The initial version of the Lexical
204 Decision Task was placed online using Qualtrics. The online task with 100 real word -
205 pseudoword pairs was promoted on a social media account of the Faculty of Psychology at
206 Chulalongkorn University, Bangkok. When interested individuals clicked on the link, they
207 were taken to the Qualtrics site and the research was explained to them in text form, at which
208 point they provided consent to continue by checking a box. Initial questions additional in the
209 survey asked about the

210

211-point self-rating of proficiency in Thai language

212

213 Next, participants read the instructions specifying that they will see pairs of words
214 comprising of a real word and a pseudoword that does not have a meaning in Thai language,
215 and that their task was to select the word in each pair that was a real word. An example item
216 and correct response was provided. The main task was presented with pairs of real and
217 pseudowords displayed in the standard default font in a legible 17pt size, with all 100 items
218 appearing on the same page on the screen. The locations of the real words and pseudowords
219 were randomized so that the real words / pseudowords did not always appear consistently on
220 either the left or right side. At the end of the task, participants were told how many they had
221 answered correctly. This feedback element appeared to make the task challenging and the initial
222 social media link was shared many times, resulting in the task being attempted 989 times over

223

224

a two-day period. At this point the task was taken offline.

# 225 2.2. Results and discussion of the online study

## 226 2.2.1. Data processing

227 The initial data set from Qualtrics contained 989 attempts. We performed data cleaning
228 to improve the quality of the dataset by removing cases with incomplete data. We also removed
229 cases completed while outside of Thailand, based on IP-addresses (as these may have included
230 non-native Thai speakers), and subsequent attempts from the same IP address (as these may
231 have indicated people completing the task more than once). Some attempts had been completed
232 in an unfeasibly short period of time. Other attempts had taken an unreasonably long time to
233 complete and may indicate participants researching the words before answering, such as
234 checking in dictionaries. Cut offs of <180 seconds and >900 seconds for task completion were
235 used as reasonable criteria for removing temporally invalid records. Of the retained cases, the
236 mean completion time was just under seven minutes (412 seconds), equivalent to completing
237 the task by spending less than 4 seconds on each choice made.

## 238 2.2.2. Demographics of the retained sample

239 Of the retained sample of 662 participants, the mean age was 32.6 years (SD = 6.8).
240 Two-thirds (66%) of the sample identified as female, 154/662 as male (23%) and the remainder
241

-reported education level was generally high, likely a
242 consequence of the online task being promoted from a university social media account. Only
243 28/662 participants (4%) reported no formal education beyond high school. The majority,
244 353/662 (53%) reported undergraduate education, and 278/662 (42%) reported graduate level
245 education. The sample, therefore, overrepresents the most highly educated section of Thai
246 society. All participants reported exposure to Thai language before the age of five, but 232
247 (35%) of them indicated that they were exposed to both Thai and other languages during that
248 period.

## 249 2.2.3. Analysis of individual items and factor analysis

250 In the next stage of analysis, the accuracy of performance from the 662 participants was
251 examined on an item-by-item basis. One item (#53) was found to have very low accuracy, and
252

253 Thai dictionaries. Consequently, responses to that item were removed from the data set. Several
254 other items appeared to produce very little variance in response, with correct responses from
255 the majority of participants. We examined this in both the full sample of 662 participants, and
256 the subsample of 28 participants with low education levels (high school or lower). Items were
257 removed from the data set if accuracy in the full sample was > 97.5%. However, items were
258 retained if scored as < 94% correct in the lower-education subsample. This allowed us to retain
259 items that may be challenging to less educated participants. This resulted in the removal of 31
260 further items. This pruning was necessary as items with very low variance would not be
261 psychometrically useful and would not be appropriate for factor analyses. There were also 6
262 items that were answered at < 50% accuracy and were removed at this stage. As the chance
263 guessing rate is 50%, they may indicate items in which the pseudoword was perceived as more
264 likely correct than the real word. As such, they reflect the interpretation of the pseudoword
265 more than the target word and lack face validity as a test of vocabulary. Together, this totaled
266 removal of 38 of the original items, leaving 62 items with a reasonable level of variance.
267 Next, the factor structure of the data set from the 662 participants on the 62 lexical-
268 decision items was examined. Because the responses to individual items by individual
269 participants have Boolean values expressing whether they were answered correctly or not (1 =
270 true, 0 = false), Pearson correlations cannot be used in exploratory factor analysis. Instead,
271 polychoric correlations are recommended [29]. The use of polychoric correlations for the
272 Boolean data of observed variables assumes that there is a continuous and normally distributed
273 scale underlying each of the observed variables. This assumption is reasonable in the sense that
274 the vocabulary knowledge of individuals is widely conceptualized as existing on a continuum
275 (e.g., [30]). Polychoric correlation coefficients range from 0 to 1, in which the values closer to
276 0 indicate weaker association and those closer to 1 indicate stronger association.
277 Next, a 62 x 62 matrix of the polychoric correlation values was submitted to exploratory
278 factor analysis. The estimation method used was the iterated principal factor method with the
279 prior communality for each variable set to the squared multiple correlation with all other
280 variables. In addition, the number of factors to be extracted was set to one because all the items
281 are intended to measure only the breadth of the mental lexicon. The scree plot of eigenvalues
282 confirmed the specification of a single factor. Finally, it is thought that loadings < .3 do not
283 meet the minimal level for inclusion within a factor [31]. With that threshold, 20 additional
284 items were removed from the scale. Consequently, the final Thai Lexical Decision Task had

285

286

42 remaining items. Those items, along with their factor loadings, are shown in Table 1.

# 287 Table 1. Items included in the final version of the Thai Lexical Decision Task based on

288 **analyses of the online study (*n =* 662)**

Item Number

Word Pair	English translation of real word

Accuracy	Factor Loading

1 Head	98.8	.78

2
Ladle	97.9	.49

3
Lips	97.6	.93

4
Snake	98.9	.57

5 Bird	98.2	.81

6	Female bird	71.9	.39

7 King	92.7	.64

8	Royal barge	94.1	.52

9 Crab	97.1	.55

10	Casket	93.2	.63

11	Sea, ocean	95.5	.47

12 Horse	88.8	.42

13 Saliva	91.5	.66

14 Day	89.3	.64

| 15 | 'Ulll'W - �flll | Rat | 96.7 | .64 |
| --- | --- | --- | --- | --- |
| 16 | 't!fl - \llllll | Ambassador | 97.1 | .42 |
| 17 |  | Sun | 63.6 | .51 |
| 18 |  | Silver | 95.9 | .64 |
| 19 |  | Parentheses | 92.1 | .48 |
| 20 |  | Snake | 97.1 | .42 |
| 21 | fl'J - °H�lHllJ | Chisel | 97.6 | .51 |
| 22 |  | Forehead | 76.3 | .46 |
| 23 | *V* < � UWl'l'll�'J - 11:!ll\U | Type of drum | 93.4 | .64 |
| 24 |  | Buffalo | 93.7 | .46 |
| 25 | 'Vl�lll'il - fll'all� | Stars | 73.0 | .35 |
| 26 |  | Gold | 85.2 | .61 |
| 27 | lll°H - IJl | Sword | 95.3 | .48 |
| 28 |  | Snake | 97.1 | .61 |
| 29 | tl�nîi - ,mm | Question mark | 88.7 | .40 |
| 30 |  | Goad stick | 86.4 | .51 |
| 31 | ll'all'll - lll,JlJ | Lotus | 95.3 | .66 |
| 32 |  | Moon | 92.7 | .42 |
| 33 | m�� -1Ï'll1Îlm | Middle finger | 97.1 | .73 |
| 34 | VllÛ-mm | Tree | 83.4 | .33 |
| 35 |  | Crown | 90.0 | .58 |
| 36 |  | Cockatoo | 95.6 | .53 |
| 37 |  | Crystal, glass | 69.6 | .39 |
| 38 |  | Forest | 97.0 | .57 |
| 39 | W-1'11 - flflÎÎ | Older sister | 98.2 | .53 |

| 40  | Ship | 90.3 | .63 |
| --- | --- | --- | --- |
| 41   | Ring | 94.1 | .69 |
| 42  | Cat | 93.5 | .32 |

289 The correct word in each pair is indicated in bold.

## 290 2.2.4. Final version of the Thai Lexical Decision Task

291 Those 42 items were used to calculate a Thai Lexical Decision Task total score by
292 summation. Psychometric properties of that total score are shown in Table 2. The internal
293 consistency of the whole scale was estimated with Kuder-Richardson 20 (KR-20) which is
294 t used with dichotomous scores [32]. This was found to
295 ness and kurtosis are not included in the table as it
296 also contains data from a separate, smaller sample (described in Section 3.), and ways to
297 interpret non-normality vary by sample sizes [33]. However, in the current (online) sample the
298 absolute skewness value of -2.24 suggests substantial negative skewing of the data distribution,
299 which is also shown by the central tendency (mean = 38, median = 39) being close to the
300 maximum (42). This likely reflects the high education level of the sample. The distribution was
301 also highly leptokurtic, with an absolute value of 10.1, which would also indicate non-normal
302 distribution, probably also linked to the highly skewed education level of the sample. Given
303 the highly non-normal distribution, non-parametric correlations were used to examine
304 associations with demographic factors. Gender was dichotomized for this analysis (male or
305 female). Thai Lexical Decision Task total scores had moderate associations with education
306

level and self-
307 ssociation with age (based on interpretation
308 thresholds from [34]). There was a very small, but significant, correlation of task performance
309 with gender, indicating slightly better performance by female participants.

310

311

# Table 2. Psychometric properties and correlates of the Thai Lexical Decision Task (42

312 **items) score distributions from the online and interview samples**

|  | | Online sample | Interview sample |
| --- | --- | --- | --- |
| N = |  | 662 | 90 |
| KR-20 |  | 0.81 | 0.88 |
| Mean |  | 38.32 | 32.58 |
| Median |  | 39.00 | 34.00 |
| Range |  | 15-42 | 17-42 |
| Coefficient of variance |  | 10% | 21% |
| Correlation | Education level | 0.18 ^a^ | 0.42 ^a,^ *** |
|  | Proficiency | 0.20 ^a,^ *** | 0.46 ^a,^ *** |
|  | Age | 0.28 ^a,^ *** | 0.39*** |
|  | Gender | 0.09 ^a,^ * | .011 |

313 *= *p<* .050, *** = *p <* .001, ^a^ = self-rated ability in
314 Thai language.

## 315 2.2.5. Summary of the online study

316 To summarize, the findings from the online study were used to prune an original set of
317 100 items to a set likely to perform well as a single scale of crystalized vocabulary knowledge.
318 Although the sample used was large, it also contained an overrepresentation of highly educated
319 participants. Nevertheless, we were able to select a set of 42 items which were challenging to
320 many participants and loaded onto a single factor, assumed to represent vocabulary knowledge.
321 One question raised is whether this scale will perform well when applied to a more
322 educationally diverse sample; that issue is dealt with in the next studies. Although the devised
323 42-item scale has obvious face validity as a measure of vocabulary knowledge in Thai, its
324 psychometric validity remains to be demonstrated formally. Thus, a further question addressed

| 325 | in the next study is whether scores on the Thai Lexical Decision Task are associated with |
| --- | --- |
| 326 | performance on more-established vocabulary assessments. |
| 327 |  |
| 328 | **3. Study 2: Task validity with an interviewed sample** |
| 329 | **3.1. Method** |
| 330 | ***3.1.1. Aims, study design, and hypotheses*** |
| 331 | The aims of this study were to examine the validity and psychometric properties of the |
| 332 | 42-item Thai Lexical Decision Task in greater detail, and in a more-educationally diverse |
| 333 | sample. To assess concurrent validity, a new sample of participants was recruited and assessed |
| 334 | one-to-one with a range of Thai-language based measures. Validation was attempted by cross- |
| 335 | sectional analysis of the correlations between the Thai Lexical-decision Task and other existing |
| 336 | tests of Thai vocabulary. It was thus hypothesized that there would be sizable positive |
| 337 | correlations between Thai Lexical Decision Task scores and other measures of vocabulary. It |
| 338 | was also hypothesized that, as a measure or acquired crystallized ability, the positive |
| 339 | associations found in the online study (described in Section 2.0.) of task performance with age, |
| 340 | education, and proficiency would be replicated. |
| 341 | ***3.1.2. Participants*** |
| 342 | A sample of 90 adult participants was recruited through advertisements on social media |
| 343 | and personal contacts. The recruitment period was the same as Study 1. The mean age of the |
| 344 | sample was 36.0 (*SD* = 16.7, range 18-68). The majority were men (*n =* 62, 69%), with 24 |
| 345 | women (*n=* 24, 27%), with the remainder identifying as other genders (*n=* 4, 4%). The sample |
| 346 | was educationally diverse, with the total years of formal education completed ranging from 4 |
| 347 | to 29; however, the mean was still rather high at 17.6 years (*SD* = 3.4). In terms of education |
| 348 | level completed, 36/90 (40%) had high school or less as their highest completed level (compare |
| 349 | this with the 4% reported in the online study described in Section 2.0.). Indeed, the current |

350 (interview) sample had a statistically significant lower education level than the previous
351 (online) sample, X^2^ = 165.75, *p*  *V* = .469.
352 Although Thai is the official and most common language spoken in Thailand, the
353 country is multicultural and other languages are frequently spoken, including English, Chinese,
354 and indigenous tribal languages. Nevertheless, understanding Thai language was a study
355 inclusion criterion. During the assessment, all participants were asked to rate themselves on
356 proficiency in Thai language on a 5-point scale from very low to very high and asked about
357 their exposure to Thai before the age of 5. The majority of the participants (*n=* 89, 99%) were
358 exposed to Thai language before the age of 5. The one exception nevertheless rated themselves
359 as very high proficiency as an adult. In contrast, one participant rated their proficiency in Thai
360 language as very low (a bilingual Thai and Chinese participant). The majority, though, rated
361 their skill in Thai-

## 362 3.1.3. Assessments

363 To examine concurrent validity of the Thai-lexical Decision Task, we sought out
364 established crystalized-cognitive measures of Thai vocabulary for adults. However, few such
365 tests exist that could be considered already validated for use as criterion variables. The tests
366 included were the best available options.
367 The main measure employed were verbal fluency tasks. In such tasks, participants are
368 required to say as many words as possible from a specified set, within a time limit of one
369 minute. Various fluency tasks have been validated within the Thai population, mainly through
370 neuropsychological studies. This is because they have been used as individual tests with
371 patients with known cognitive impairments (e.g., [35]) or they have been used in computerized
372 assessment studies (e.g., [36]), or they have been included as subtests in screening tools for
373 cognitive impairments (e.g., [37]).
374 Verbal fluency tasks tend to measure both fluid (e.g., executive) and crystalized (i.e.,
375 language) ability [14]. Nevertheless, these tests are useful as criterion measures as they have
376 been included in so many cognitive screens validated in Thailand that the soundness of their
377 psychometrics properties is established. In addition, in healthy participants, as in the current
378 study, performance appears to be limited mainly by variation in language knowledge, rather
379 than executive function [38].
380 Several forms of verbal fluency task are available in Thai, differing on the set specified
381 to the participant. We used six such tasks, one trial for each type. One type used was category
382 fluency. This requires participants to produce words constrained by a semantic category. The
383 categories we used were all included in previous studies in Thailand 1) Animals [35,37,39
384 41], 2) Fruits [35], and 3) First Names [41,42].
385 We also included phonemic fluency trials. In such tasks, the limiting factor given to the
386 participants is that words should begin with a particular phoneme. We used fluency for , , and
387 characters of the Thai writing script. These Thai characters have been used in phonemic
388 fluency tasks in numerous validity and task-development studies [3537,39].
389 We recorded one minute of responses for each of the six trials but distinguished within
390 the data between the number of words reported in the first 30 and subsequent 30 seconds. This
391 was achieved simply by having the researcher draw a line across the page at the 30 second
392 point. We did this because it is thought that relatively automatic lexical memory searching is
393 the principal skill indexed by performance in the first 30 seconds, later performance indexes
394 more executive cognitive control, e.g., search strategy [43,44]. As we expected our lexical-
395 decision task to assess breath of lexical stores (not executive control), we additionally
396 hypothesized that correlations should be higher for scores from the first 30 seconds, than the
397 subsequent 30 seconds.
398 We also used the Boston Naming Test, which is part of the Boston Diagnostic Aphasia
399 Examination and is thus designed for neuropsychological use [45]. It involves patients being
400 presented with drawings of objects and they are asked to name them. Although this is a clinical
401 tool, a validated short form is available in Thai, and even healthy controls produced a range of
402 scores without a substantial ceiling effect, suggesting that the test potentially measures
403 vocabulary in non-clinical populations [46]. That version contains images of five low-
404 frequency, and five medium-frequency objects. The total score range is 0 (worst) to 10 (best).
405 The Thai Lexical Decision Task with 42 items, as developed in the online study
406 described in Section 2.0. was employed. However, as this was one-to-one data collection, a
407 paper copy of the test was provided, and participants asked to circle the real word in each word
408 pair. This study was started simultaneously with the online study, but continued for a longer
409 period, and so the full initial 100-item task was employed, the same set as in the online study.
410 However, the analysis is limited to scores from the final 42-item version.

## 411 3.1.4. Procedure

412 The procedures applied, including taking written consent, were approved by the local
413 ethics committee, as described in Section 2.1.4. All participants were recruited through
414 advertisements or personal contacts, and provided written, informed consent. All were assessed
415 face-to-face in a one-on-one interview setting.

| 416 |  |
| --- | --- |
| 417 | **3.2. Results and discussion of the interview study** |
| 418 | ***3.2.1. Psychometric properties of the total score*** |
| 419 | The psychometric properties of the 42-item Thai Lexical Decision Task in the 90 |
| 420 | participants interviewed face-to-face are shown in Table 2, alongside the equivalent statistics |
| 421 | from Study 1. As in the previous study, the scale had good internal consistency as estimated |
| 422 | with KR-20. In contrast to the previous study, in the interview sample the data distribution |

423 appeared to be normal, with skewness (*z* = 2.40) and kurtosis (*z* = 1.15) both within limits for
424 a normal distribution [33]. Normality of data distribution was confirmed by examination of the
425 Q-Q plot. Visual analysis of the data distribution did suggest that it was somewhat negatively
426 skewed, but considerably less than in the previous online study. This is also reflected in the
427 higher degree of variation in task performance scores in the current sample, compared to the
428 Study 1 sample, based on the much higher coefficient of variance.
429 This may be because of the increased diversity of educational background in the interview
430 sample; indeed, the measures of central tendency for Thai Lexical Decision Task scores are
431 around 5 points lower, reducing the previously observed ceiling effect. This may also be why
432 the association between task scores and education was of a larger magnitude in this study, with
433 [34]. Similarly, there was a larger positive association with age,
434 likely also linked to the greater age diversity in this sample.
435 The coefficient of variance was relatively large at 21%, which would indicate that the
436 scale provides a reasonable amount of differentiation of scores across participants for it to be
437 used as a measure of individual differences in performance. In fact, this is a higher value that
438 reported for the English-language Spot-the-Word Test in non-clinical samples which had a
439 coefficient of only 8% in a New Zealand study [47] and 9% in a USA-based study [48], and
440 16% in a UK-based study [49]. This may suggest that the scale could function well as a tool to
441 measure individual differences, in which the ability to rank participants is an important feature
442 of task reliability [50].
443 All cognitive tests scores were correlated with age of the participants, which is
444 understandable considering the age-diversity of the sample (ages 18-68). Age effects on
445 cognition are variable in adulthood, with more-fluid abilities tending to decline with age and
446 more-crystallized abilities tending to increase with age [5]. Our cognitive tests scores showed
447 the expected pattern, with lexical decision scores, the presumptive crystalized measure,
448 showing a positive association, and the other tasks, thought to contain fluid processing aspects,
449 tending to show negative associations. To negate these potentially confounding effects, age-
450 adjusted data distributions were created for all cognitive measures by regressing test scores
451 with age and storing the residuals. These age-adjusted scores were then used in later analyses.

## 452 3.2.2. Correlations of Thai Lexical Decision Task with criterion variables

453 The data distributions of the age-adjusted scores for the Thai Lexical Decision Task
454 and all measures of verbal fluency, and the Boston Naming Test did not appear to vary from
455 the normal distribution, based on analysis of skew and kurtosis nor visual inspection of
456 distributions and Q-Q plots. Therefore, parametric correlations were used to assess the

457

458

associations. These are shown in Table 3.

# 459 Table 3. Mean verbal fluency scores and Pearson correlation r values for the

460	**associations with Thai Lexical Decision Task scores**

|  | | Semantic |  | Phonemic |  |
| --- | --- | --- | --- | --- | --- |
|  |  | Mean (*SD*) | r | Mean (*SD*) | r |
| 00-30 Sec. | | 39.2 (9.4) | .51*** | 25.8 (8.2) | .40*** |
| 31-60 Secs. | | 23.1 (5.6) | .38*** | 16.3 (6.8) | .34*** |
| 00-60 Secs. | | 62.3 (13.4) | .51*** | 42.1 (13.4) | .42*** |
| 461 | Means show sum of words over 3 trials, *** *p<* .001 | | | |  |
| 462 |  | | | |  |
| 463 | The pattern of correlations indicates strong positive correlations | | | | between the Thai |

464 Lexical Decision Task and both semantic and phonemic fluency measures. The effect is
465 strongest in the first 30-second period of the task, compared to the second 30-second period.
466 This is consistent with past arguments that the first period of the task is more associated with
467 automated searching of lexical entries, compared to the subsequent period of task performance
468 in which performance variation is due to executive control factors [43,44]. Thus, of the
469 correlation r values reported, those for the 0-30 second period are more relevant for validation
470 purposes, adding further support to the validity of the Thai Lexical Decision Task as a measure
471 of vocabulary. Not shown in the table is the high positive correlation between the Thai Lexical
472 Decision Task and total verbal fluency performance (combining all fluency tasks trials), *r =*
473 .51, *p <* .001.
474 We also examined the association of the Thai Lexical Decision Task with scores on the
475 abbreviated Boston Naming Test. There was again a positive correlation between performance
476 on the two tasks, *r =* .38, *p <*.001. Also relevant to assessing validity is the high correlation
477 between Thai Lexical Decision Task scores and self-rated proficiency in the Thai language (*r*

478	*=* .46, *p<* .001).

479 To interpret the magnitude of these correlations, for validity studies of cognitive tests,
480 correlation coefficients can be qualitatively
481 [51].
482 The validity estimates in this study can therefore all be considered to range from moderate to
483 very high, depending on the measures.

## 484 3.2.3. Summary of the interview study

485 To summarize the results from this study, the reduction from 100 items in the original
486 version to the smaller 42-item Thai Lexical Decision Task appears to have produced a scale
487 with good internal consistency. This therefore replicates the finding from Study 1 of good
488 internal consistency in an independent sample. In addition, the scale produces a wide range of
489 scores indicating its potential as an individual differences measure of ability. Furthermore, it
490 can be seen as a validated measure of vocabulary, based on its associations with existing
491 measures of vocabulary in Thai.
492 The final psychometric issue is whether the Thai Lexical Decision Task can be
493 considered a reliable measure of vocabulary. As a self-completion scale, there is no issue with

| 494 | inter-rater reliability to address. Although split-half reliability could be calculated, that is a |
| --- | --- |
| 495 | rather weak estimate of reliability as it is dependent on the ordering of items. Besides which, |
| 496 | the KR-20 analysis already described indicated good internal consistency, and that is equivalent |
| 497 | to the mean of all possible spilt-half reliability coefficients [32]. The remaining question is |
| 498 | whether the Thai Lexical Decision Task has temporal stability, that is, a practice effect and |
| 499 | test-retest reliability. This is examined in Study 3. |
| 500 |  |
| 501 | **4. Study 3: Temporal stability of the Thai Lexical Decision Task** |
| 502 | **4.1. Method** |
| 503 | ***4.1.1. Aims, study design and hypotheses*** |
| 504 | The aim of this study was to assess the stability of Thai Lexical Decision Task Scores |
| 505 | over time. To this end, a prospective follow-up of a subsample of participants from the study |
| 506 | described in Section 3.0. was performed. This allowed us to examine the practice effect and |
| 507 | test-retest reliability. It was hypothesized that there would be a high correlation between |
| 508 | sessions when participants perform the Thai Lexical Decision Task on two separate occasions. |
| 509 | ***4.1.2. Participants*** |
| 510 | A subsample of 27 participants from the study described in Section 3.0 was retested. |
| 511 | The demographics from this subsample did not differ significantly from the remainder of the |
| 512 | sample described in Section 3.1.2. (mean age = 37.5, *SD* = 18.3; mean years of education = |
| 513 | 18.4, *SD* = 3.7; 67% men). |
| 514 | ***4.1.3. Procedure*** |
| 515 | The same procedure as for the previous study (described in Section 3.0) was employed, |
| 516 | with the exception that neither the consent procedure nor collection of demographic |
| 517 | information were repeated. Otherwise, the same task sequence and task rules were applied. |
| 518 | Each participant was retested in the same location as the original data location reported in the |

519 previous study, with a small number of exceptions. All were assessed by the same research
520 assistant who performed the initial interview. Retest sessions were conducted approximately 5
521 weeks after the first test session (mean gap in days = 35, *SD* = 10, range = 21 - 61).

## 522 4.1.4. Assessment tools

523 The same assessment tools as described in the previous study were used again in this

524

525

study.

# 526 4.2. Results and discussion of the temporal stability study

527 There appeared to be no obvious practice effect, the mean score at the original test
528 session was 32.2 (*SD* = 7.2) and at retest it was 31.9 (*SD* = 7.4), in fact, retest scores were
529 marginally lower than test scores. To examine the test-retest reliability, we used Pearson
530 correlation. This indicated a very strong association between scores at the initial test session
531 and the follow-up retest session, *r=* .92, *p <* .001. That value exceeds even the most stringent
532 -retest reliability [52].

## 533 4.2.1. Summary of the temporal stability study

534 To summarize this study, scores on the Thai Lexical Decision Task appear to be very

535

536

stable over time, indicating a lack of practice effect and high test-retest reliability.

# 537 5. General Discussion

538 The overall aim of this research was to develop a standardized lexical-decision task for
539 use with Thai-speaking participants. As a measure of acquired, crystalized vocabulary, the task
540 showed the expected associations with self-report variables, being positively correlated with
541 age, education level, and Thai-language proficiency. It also appears to be a psychometrically
542 sound, valid and reliable measure of vocabulary. Here we discuss in more detail some issues
543 raised by the research.

# 544 5.1. Critical evaluation of the methods and results

545 Initial data was collected using online methods. This has pros and cons. Although this
546 allowed us to collect data on a relatively large sample of almost one thousand participants, the
547 selection of participants was uncontrolled. To enhance data quality, a full third of the sample
548 had to be excluded. Nevertheless, this still left a sizable sample which allowed us to use factor
549 analysis methods to select a set of the best performing items loading onto a single latent factor,
550 presumably lexical knowledge.
551 The eventual tool produced, the Thai Lexical Decision Task, has 42 items, each a real
552 word paired with a pseudoword. This is somewhat shorter than similar tests available in other
553 languages. For example, the original English-language Spot-the-Word test had 60 word-pairs
554 [19,21] and a later version had 100 pairs [20]. Thus, the current test will produce a narrower
555 range of scores than those tests. However, doubt has been raised as to whether so many items
556 are even needed for the Spot-the-Word test to function as an estimator of crystalized
557 intelligence (its principal use). Indeed, van der Linde et al. [49] have provided evidence that a
558 reduced set of only 34 items performs better than the full set or 100 items for predicting full-
559 scale IQ scores.

# 560 5.2. Potential applications

561 One reason that prediction of IQ scores by vocabulary tests, including lexical-decision
562 tasks, is valued, is so that they can potentially be used as estimators of premorbid cognitive
563 function for neuropsychological assessment. The tests used are usually either word
564 pronunciation tasks in which phoneme-grapheme conversion would be ineffective (e.g.,
565 irregular spelled words in English), or lexical decision tasks (such as the Spot-the-Word Test).
566 Often very high correlations are observed between such lexical tasks and full-scale IQ in
567 healthy samples [6,28]. Because of this observation, regression formulas can be produced that
568 can be used to predict IQ scores, or else the lexical test can be co-normed with an IQ test (e.g.,
569 [53]). Just on that basis, the lexical task can then rapidly estimate IQ scores. In fact, lexical
570 tasks can be combined with demographic factors to produce regression-based test norms for
571 various measures of cognitive ability, even when the correlations are not so strong [54].
572 However, to be effective as estimators of premorbid ability, performance of the lexical
573 task has to be robust in the presence of disorders that would be expected to negatively impact
574 cognitive function. This is based on the previously discussed distinction between fluid
575 cognitive processes which are generally susceptible to cognitive impairment, and crystalized
576 ability which is far less likely to be impaired in the presence of neurological or psychiatric
577 disorders. Lexical decision tasks are designed to measure that crystalized ability [21] and
578 evidence suggests that lexical-en in the presence of mild
579 dementia. This has been shown with tests in various languages including English [55],
580 Portuguese [27], and Spanish [6].
581 The current test, the Thai Lexical Decision Task, could potentially be used in this role,
582 although further research would be needed to confirm that it too, as a measure of crystalized
583 ability, is robust in the presence of cognitive impairment that nevertheless impairs fluid ability.
584 Though, it seems likely that it would be. Firstly, there is no obvious reason why lexical decision
585 tasks in Thai would not show such robustness when similar tests in other languages have been
586 shown to be so. Secondly, we deliberately designed the Thai Lexical Decision Task to include
587 items that would likely hold, as this would enhance them as indices of crystalized ability. We
588 did this by using only concrete, imageable words. This was done because imageability is an
589 important factor in whether word forms are susceptible to retrograde loss in dementia [56].
590 This is not a feature of tests in other languages such as the Spot-the-Word Test [19,21] or
591 Spanish Lexical Decision Task [26].
592 It would also be useful to establish the strength of correlation with other cognitive tests.
593 IQ is often used for premorbid estimations because vocabulary is extremely closely linked to
594 general intelligence and may be the best single predictor of general cognitive ability [11,57].
595 This is a very useful association; because of the general manifold of positive test
596
597 particularly lexical measures [58], will allow some level of prediction of their performance on
598 any cognitive test.
599 Nevertheless, such lexical tests generally have their strongest statistical associations
600 with cognitive tests that have high verbal content [6,59]. As an example, the Spot-the-Word
601 Test was originally intended as a premorbid predictor of speed of semantic processing of
602 reading sentences [21], based on the strong correlation between the two different measures of
603 around *r=* .57.

# 604 5.3. Strength of validity evidence

605 At this point, it is worth considering the strength of the correlations reported in the
606 current study between Thai Lexical Decision Task scores and measures of verbal fluency and
607 picture naming (Boston Naming Test). Here, we were primarily interested in the use of
608 correlation coefficients to establish test validity. The highest r values reported in this respect
609 were a little over 0.5. Some authors have argued that values should be higher than that in
610 validity studies, while others have suggested much lower thresholds, for example, that values
611 [51,60]. In practice, the
612 threshold used should reflect the similarity of the measures, the specific hypotheses made, etc.
613 We feel that the current findings are indicative of the validity because the verbal fluency
614 tasks and the Boston Naming Test were both rather different in format to the lexical decision
615 task. As such, very high correlations could not be reasonably anticipated.
616 Furthermore, the relative sizes of the correlation coefficients for verbal fluency were
617 consistent with our hypothesis that performance in the first half of the tasks would be a better
618 indicator of lexical knowledge than performance in the second half. In fact, our results provide
619 additional support to previous suggestions that the first 30 seconds of performance of verbal
620 fluency tasks are more dependent on breadth of the lexicon, compared to the subsequent time
621 spent on individual trials [43,44].
622 Given the simplicity of the Thai Lexical Decision Task, and its minimal involvement
623 of cognitive control for completion, it appears to have substantial face validity. We therefore
624 argue that the validity of the task in question as a measure of crystalized vocabulary knowledge
625 is practically established.
626 We would also note that the values observed are in fact slightly higher than those
627 typically found between another widely used measure of crystalized ability in English, the
628 National Adult Reading Test [7], and the same tests used in this study, namely the verbal
629 fluency and the Boston Naming Test [59]. Another potential application of the Thai Lexical
630 Decision Task is as a brief estimator of verbal intelligence. Full intelligence tests can be very
631 time consuming and impractical in research. Brief estimators of intelligence are therefore
632 useful.

| 633 | **5.4. Conclusion** |
| --- | --- |
| 634 | We present a brief lexical decision task that can be used to assess crystalized vocabulary |
| 635 | knowledge in Thai. The test appears to be an internally consistent, valid, and reliable measure |
| 636 | of such knowledge. The Thai Lexical Decision Task is free to use in research without cost, and |
| 637  638 | can be downloaded from: https://gpluck.co.uk/Tests/ |
| 639  640 | **Acknowledgements:** We would like to thank Nutvara Patitus, Kamorichan Osthananda, |
| 641 | Panyada Khongthong, and Saranwat Jetsawangsri for assistance with data collection, and Fei |
| 642 | Gu for assistance with conducting the factor analysis. This research did not receive any specific |
| 643 | grant from funding agencies in the public, commercial, or not-for-profit sectors. |

644

# 645 References

646 1.	Cattell RB. The theory of fluid and crystallized general intelligence checked at the 5-6
647 year-old level. Brit J of Edu Psychol. 1967;37: 209224. doi:10.1111/j.2044-
648 8279.1967.tb01930.x
649 2.	Baddeley A. The episodic buffer: a new component of working memory? Trends in

650	Cognitive Sciences. 2000;4: 417423. doi:10.1016/S1364-6613(00)01538-2

651 3.	Wechsler D. Wechsler Abbreviated Scale of Intelligence. 2012. doi:10.1037/t15170-000
652 4.	Carlsson M, Dahl GB, Öckert B, Rooth D-O. The Effect of Schooling on Cognitive
653 Skills. Review of Economics and Statistics. 2015;97: 533547.
654 doi:10.1162/REST_a_00501
655 5.	Craik FIM, Bialystok E. Cognition through the lifespan: mechanisms of change. Trends

656	in Cognitive Sciences. 2006;10: 131138. doi:10.1016/j.tics.2006.01.007

657 6.	Pluck G, Ruales-Chieruzzi CB. Estimation of premorbid intelligence and executive
658 cognitive functions with lexical reading tasks. Psychology & Neuroscience. 2021;14:
659 358377. doi:10.1037/pne0000264
660 7.
661 Using the New Adult Reading Test. Cortex. 1978;14: 234244. doi:10.1016/S0010-
662 9452(78)80049-5
663 8.	Kyllonen P, Anguiano Carrasco C, Kell H. Fluid Ability (Gf) and Complex Problem
664 Solving (CPS). J Intell. 2017;5: 28. doi:10.3390/jintelligence5030028
665 9.	Duncan J, Burgess P, Emslie H. Fluid intelligence after frontal lobe lesions.

666	Neuropsychologia. 1995;33: 261268. doi:10.1016/0028-3932(94)00124-8

667 10. Fedorenko E, Varley R. Language and thought are not the same thing: evidence from
668 neuroimaging and neurological patients: Language versus thought. Ann NY Acad Sci.
669 2016;1369: 132153. doi:10.1111/nyas.13046
670 11. Schipolowski S, Wilhelm O, Schroeders U. On the nature of crystallized intelligence:
671 the relationship between verbal ability and factual knowledge. Intelligence. 2014;46:
672 156168. doi:10.1016/j.intell.2014.05.014
673 12. Li Y, Beaty RE, Luchini S, Hu W, Kenett YN. The role of semantic memory networks
674 in crystallized intelligence and creative thinking ability. Learning and Individual
675 Differences. 2024;111: 102426. doi:10.1016/j.lindif.2024.102426
676 13. Gansler DA, Varvaris M, Swenson L, Schretlen DJ. Cognitive estimation and its
677 assessment. Journal of Clinical and Experimental Neuropsychology. 2014;36: 559568.
678 doi:10.1080/13803395.2014.915933
679 14. Aita SL, Beach JD, Taylor SE, Borgogna NC, Harrell MN, Hill BD. Executive,
680 language, or both? An examination of the construct validity of verbal fluency measures.
681 Applied Neuropsychology: Adult. 2019;26: 441451.
682 doi:10.1080/23279095.2018.1439830

Gao Z, Zheng L, Chiou R, Gouws A, Krieger-Redwood K, Wang X, et al. Distinct and common neural coding of semantic and non-semantic control demands. NeuroImage. 2021;236: 118230. doi:10.1016/j.neuroimage.2021.118230

| 683 | 15. |
| --- | --- |
| 684 |  |
| 685 |  |
| 686 | 16. |
| 687 |  |
| 688 |  |
| 689 | 17. |
| 690 |  |
| 691 |  |
| 692 |  |
| 693 |  |
| 694 | 18. |
| 695 |  |
| 696 | 19. |
| 697 |  |
| 698 |  |
| 699 | 20. |
| 700 |  |
| 701 | 21. |
| 702 |  |
| 703 | 22. |
| 704 |  |
| 705 |  |
| 706 | 23. |
| 707 |  |
| 708 |  |
| 709 | 24. |
| 710 |  |
| 711 |  |
| 712 | 25. |
| 713 |  |
| 714 | 26. |
| 715 |  |
| 716 |  |
| 717 |  |
| 718 | 27. |
| 719 |  |
| 720 |  |
| 721 |  |
| 722 |  |

Steger D, Schroeders U, Wilhelm O. On the dimensionality of crystallized intelligence: A smartphone-based assessment. Intelligence. 2019;72: 7685. doi:10.1016/j.intell.2018.12.002

Ullman MT, Corkin S, Coppola M, Hickok G, Growdon JH, Koroshetz WJ, et al. A Neural Dissociation within Language: Evidence that the Mental Dictionary Is Part of Declarative Memory, and that Grammatical Rules Are Processed by the Procedural System. Journal of Cognitive Neuroscience. 1997;9: 266276. doi:10.1162/jocn.1997.9.2.266

Lehrl S. Mehrfachwahl-Wortschatz-Intelligenztest B (MWTB) [Multiple-choice vocabulary intelligence test]. Balingen, Germany: Spitta; 1971.

Word test: A robust estimate of verbal intelligence based on lexical decision. British J Clinic Psychol. 1993;32: 5565. doi:10.1111/j.2044-8260.1993.tb01027.x

Baddeley A, Crawford J. Spot the Word. 2nd edition. Oxford: Pearson Assessment; 2012.

Baddeley A, Emslie H, Nimmo-Smith I. Speed and Capacity of Language Processing (SCOLP) Test Manual. Bury St Edmunds: Thames Valley Test Company; 1992.

Guo C, Tsegaye A, Arató J, Alexander Logemann HN. The role of attention, inhibition and statistical learning in Chinese character recognition by novices. Current Research in Behavioral Sciences. 2021;2: 100012. doi:10.1016/j.crbeha.2020.100012

controlled semantic memory retrieval. Mem Cogn. 2023;51: 11451158. doi:10.3758/s13421-022-01388-x

Mulligan NW, Peterson D. Attention and implicit memory in the category-verification and lexical decision tasks. Journal of Experimental Psychology: Learning, Memory, and Cognition. 2008;34: 662679. doi:10.1037/0278-7393.34.3.662

Brysbaert M. Lextale_FR A Fast, Free, and Efficient Test to Measure Language Proficiency in French. PSYCHOL BELG. 2013;53: 23. doi:10.5334/pb-53-1-23

Instituto de Neurociencias, Universidad San Francisco de Quito, Cumbayá, Ecuador, Pluck G, The University of Sheffield, United Kingdom of Great Britain and Northern Ireland. A lexical decision task to measure crystallized-verbal ability in Spanish. RLP. 2020;52. doi:10.14349/rlp.2020.v52.1

Serrao VT, Brucki SMD, Campanholo KR, Mansur LL, Nitrini R, Miotto EC. Performance of a sample of patients with Mild Cognitive Impairment (MCI),

measure of pre-morbid intelligence. Dement neuropsychol. 2015;9: 265269. doi:10.1590/1980-57642015dn93000009

723 28. Almkvist O, Adveen M, Henning L, Tallberg IM. Estimation of premorbid cognitive
724 function based on word knowledge: The Swedish Lexical Decision Test (SLDT).
725 Scandinavian J Psychology. 2007;48: 271279. doi:10.1111/j.1467-9450.2007.00575.x
726 29. Barendse MT, Oort FJ, Timmerman ME. Using Exploratory Factor Analysis to
727 Determine the Dimensionality of Discrete Responses. Structural Equation Modeling: A
728 Multidisciplinary Journal. 2015;22: 87101. doi:10.1080/10705511.2014.934850
729 30. Henriksen B. Three dimensions of vocabulary development. Stud Second Lang Acquis.

730	1999;21: 303317. doi:10.1017/S0272263199002089

731	31. Hair JF, Black WC, Babin BJ, Anderson RE, Tatham RL. Multivariate data analysis.

732	Hampshire, UK: Cengage Learning; 2019.

733	32. Cronbach LJ. Coefficient Alpha and the Internal Structure of Tests. Psychometrika.

734	1951;16: 297334. doi:10.1007/BF02310555

735 33. Kim H-Y. Statistical notes for clinical researchers: assessing normal distribution (2)
736 using skewness and kurtosis. Restor Dent Endod. 2013;38: 52.
737 doi:10.5395/rde.2013.38.1.52
738 34. Gignac GE, Szodorai ET. Effect size guidelines for individual differences researchers.
739 Personality and Individual Differences. 2016;102: 7478.
740 doi:10.1016/j.paid.2016.06.069
741 35. Muangpaisan W, Intalapaporn S, Assantachai P. Digit span and verbal fluency tests in
742 patients with mild cognitive impairment and normal subjects in Thai-community. J Med
743 Assoc Thai. 2010;93: 224230.
744 36. Kantithammakorn P, Punyabukkana P, Pratanwanich PN, Hemrungrojn S, Chunharas C,
745 Wanvarie D. Using Automatic Speech Recognition to Assess Thai Speech Language
746 Fluency in the Montreal Cognitive Assessment (MoCA). Sensors. 2022;22: 1583.
747 doi:10.3390/s22041583
748 37. Munthuli A, Vongsurakrai S, Anansiripinyo T, Ellermann V, Sroykhumpa K, Onsuwan

749

C, et al. Thammasat-NECTEC-

750

751 43rd Annual International Conference of the IEEE Engineering in Medicine & Biology
752 Society (EMBC). Mexico: IEEE; 2021. pp. 690694.
753 doi:10.1109/EMBC46164.2021.9630779
754 38. Whiteside DM, Kealey T, Semla M, Luu H, Rice L, Basso MR, et al. Verbal Fluency:
755 Language or Executive Function Measure? Applied Neuropsychology: Adult. 2016;23:
756 2934. doi:10.1080/23279095.2015.1004574

757	39.

758 Cognitive Examination III. Psychiatry Investig. 2016;13: 571.
759 doi:10.4306/pi.2016.13.5.571
760 40.
761 Cognitive Examination as a mild cognitive impairment and dementia screening test.
762 Psychogeriatrics. 2019;19: 340344. doi:10.1111/psyg.12417
763 41. Heaps J, Valcour V, Chalermchai T, Paul R, Rattanamanee S, Siangphoe U, et al.
764 Development of normative neuropsychological performance in Thailand for the
765 assessment of HIV-associated neurocognitive disorders. Journal of Clinical and
766 Experimental Neuropsychology. 2013;35: 18. doi:10.1080/13803395.2012.733682
767 42.
768 new neuropsychological tests designed to minimize cultural bias in the assessment of
769 HIV-1 seropositive persons: A WHO study. Archives of Clinical Neuropsychology.
770 1993;8: 123135. doi:10.1093/arclin/8.2.123
771 43. Joanette Y, Goulet P, Le Dorze G. Impaired word naming in right-brain-damaged right-
772 handers: Error types and time-course analyses. Brain and Language. 1988;34: 5464.
773 doi:10.1016/0093-934X(88)90124-1
774 44. Gordon JK, Young M, Garcia C. Why do older adults have difficulty with semantic
775 fluency? Aging, Neuropsychology, and Cognition. 2018;25: 803828.
776 doi:10.1080/13825585.2017.1374328
777 45. Kohn SE, Goodglass H. Picture-naming in aphasia. Brain and Language. 1985;24: 266

778	283. doi:10.1016/0093-934X(85)90135-X

779 46. Aniwattanapong D, Tangwongchai S, Supasitthumrong T, Hemrunroj S,
780 Tunvirachaisakul C, Tawankanjanachot I, et al. Validation of the Thai version of the
781 short Boston Naming Test (T-
782 cognitive impairment: clinical and biomarker correlates. Aging & Mental Health.
783 2019;23: 840850. doi:10.1080/13607863.2018.1501668
784 47. Barker-Collo S, Bartle H, Clarke A, van Toledo A, Vykopal H, Willetts A. Accuracy of
785 the National Adult Reading Test and Spot the Word estimates of premorbid intelligence
786 in a non-clinical New Zealand sample. New Zealand Journal of Psychology. 2008;37:
787 5361.
788 48. Yuspeh RL, Vanderploeg RD. Spot-the-Word: A Measure for Estimating Premorbid
789 Intellectual Functioning. Archives of Clinical Neuropsychology. 2000;15: 319326.
790 doi:10.1093/arclin/15.4.319
791 49. Van Der Linde I, Horsman L, Bright P. The validity of abbreviated forms of the
792 National Adult Reading Test and Spot-the-Word 2 for estimating full-scale IQ.
793 Neuropsychological Rehabilitation. 2022;32: 25342543.
794 doi:10.1080/09602011.2021.1958874
795 50. Hedge C, Powell G, Sumner P. The reliability paradox: Why robust cognitive tasks do
796 not produce reliable individual differences. Behav Res. 2018;50: 11661186.
797 doi:10.3758/s13428-017-0935-1
798 51. Swank JM, Mullen PR. Evaluating Evidence for Conceptually Related Constructs Using
799 Bivariate Correlations. Measurement and Evaluation in Counseling and Development.
800 2017;50: 270274. doi:10.1080/07481756.2017.1339562
801 52. Vilagut G. Test-Retest Reliability. In: Michalos AC, editor. Encyclopedia of Quality of
802 Life and Well-Being Research. Dordrecht: Springer Netherlands; 2014. pp. 66226625.
803 doi:10.1007/978-94-007-0753-5_3001
804 53. Wechsler D. Test of Premorbid Functioning (TOPF). San Antonio, TX: The
805 Psychological Corporation; 2011.
806 54. Testa SM, Winicki JM, Pearlson GD, Gordon B, Schretlen DJ. Accounting for
807 estimated IQ in neuropsychological test performance with regression-based techniques.
808 J Int Neuropsychol Soc. 2009;15: 10121022. doi:10.1017/S1355617709990713
809 55.
810 premorbid measures of intelligence. British J Clinic Psychol. 2006;45: 453464.
811 doi:10.1348/014466505X71245
812 56. Cuetos F, Arce N, Martínez C, Ellis AW. Word recognition in A
813 Effects of semantic degeneration. Journal of Neuropsychology. 2017;11: 2639.
814 doi:10.1111/jnp.12077
815 57. Jensen AR. Vocabulary and general intelligence. Behav Brain Sci. 2001;24: 11091110.

816	doi:10.1017/S0140525X01280133

817 58. Pluck G, Cerone A. A demonstration of the positive manifold of cognitive test inter-
818 correlations, and how it relates to general intelligence, modularity, and lexical
819 knowledge. In: Fitch T, Lamm C, Leder H, Teßmar-Raible K, editors. Proceedings of
820 the 43rd Annual Conference of the Cognitive Science Society. 2021. pp. 30823088.
821 59. Schretlen DJ, Buffington ALH, Meyer SM, Pearlson GD. The use of word-reading to

822

823	Neuropsych Soc. 2005;11. doi:10.1017/S1355617705050939

824	60. Sheperis C, Drummond RJ, Jones KD. Assessment procedures for counselors and

825	helping professionals. Ninth edition. Boston: Pearson; 2020.

826

827

Response to Reviewers

Dear Editor,

Below we provide a response to the comment we received in the decision letter for the previous submission {ID: PONE-D-25-11789).

**Editor 1**

1. Thank you very much for submitting your manuscript to PLOS ONE, and for responding to our recent requests regarding your submission. After careful evaluation, we have decided that your submission does not meet our publication criteria and must be rejected.

Based on our evaluation of your manuscript and the information you provided, we do not feel that your submission meets our ethical requirements for human subjects research submissions. PLOS ONE requires that research meets ail applicable standards for the ethics of experimentation and research integrity ([http://journals.plos.org/plosone/s/human-subjects-research).](http://journals.plos.org/plosone/s/human-subjects-research)) We reserve the right to reject any submission that does not meet our internai ethical standards, which in some cases are more stringent than local ethical standards.

We recognize that you obtained ethical approval from your local ethics committee after you began the study: your ethics approval document is dated March 9, 2023, while participants were recruited beginning in March 3, 2023. For human subjects research, PLOS ONE requires that authors obtain ethics committee approval before beginning the study, and we do not accept retrospective ethical approval.

As a result of these concerns we cannot consider the manuscript for publication at PLOS ONE. 1 am sorry that we cannot be more positive on this occasion, but hope that you understand the reasons for this decision.

**We would like to clarify that the corresponding author had made a typographical mistake in specifying the participant recruitment period in the manuscript. The corresponding author (Suphasiree Chantavarin) had emailed to ask the first author (Graham Pluck), who has access to the participant recruitment information, and Dr. Graham had replied that the recruitment period was between 13/03/2023-08/03/2024. However, when entering this information into the manuscript, the Dr. Suphasiree mistyped 13/03/2023 as 3/03/2023. Below we provide screenshots ofthis email communication between Dr. Suphasiree and Dr. Graham.**

**We have corrected this mistake in the revised manuscript (p.8, Line 167).**

**Since this mistake was due to a typographical error rather than a breach in ethical standards, we humbly request the editor to re-evaluate our submission. We apologize for the inconvenience caused.**

X < >

Fw: Your Submission CRBEHA-0-2-4-00112

i	...

,

1

*i)*	Thaile...dean.pdf	PONE-S-25-15325.pdf	S.creen	62741.png

**f))** Vou

OK,l'llgoaheadandsubmititthen!	.i.�.iyi!U�mhn-rfSuphasireeChantavann,PhD.Lecturer,mdHeadoftheCognitiveP:iychologylve.a • FarultyofP...	Wed 3/5/202� 12:56 f'M

.SuphasireeChantavarin

To:0g.c.pluck

Q +--, Reply ¾-, Reply all *rt* Forward c2i (;;] ...

Frilf7/20254:3.2PM

=----· ==

��--=�

Hi ah

The lexical decision manuscript was sent back from PloS ONE because they requested additional information about human subjects data collection (screenshot attached).

They asked us to specify the **day, month, and year** of the **start and end** of the recruitment period for this study (e.g., duration of data collection). Do you have the info regarding this?

X < >

Fw: Your Submission CRBEHA-0-24-00112

i*'l)	�* Th.ai Le...dean.pdf Q PONE-S-25-15325.pdf	·screen�..62741.png	·	1

■

**1=-----=-------------•w;---------··-------..,..	1**

Pluck<[g.c.pluclc@gmail.com](mailto:g.c.pluclc@gmail.com)>

To: esuphasireeChantavarin

Q '(---, Reply +., Reply all *r'* Forward '2i {ru •••

Fri3{7/202S.S.:22PM

13/03/2023 - 08/03/2024

�	Pluck, B.A., B.Sc., Ph.D., AFBPsS.

Chulalongkorn University, Faculty of Psychology, 8th Floor,

Borommaratchachonnani Srisattaphat Building, 254 Phayatbai Road,

Bangkok, Thailand 10330

hnP.·//[www.](http://www/)�	bJ!P.s://[www.instagram.com/J;!lucklab/](http://www.instagram.com/J%3B!lucklab/) hnps://twitter com/DrGPl11ck

**1**

| X < >  Fw: Your Submission CRBEI-IA-0-24-00112  **_1_***ffe* **-**�T**-**hai**-**le..**-**d. ean**-**.pdf**---**PON**-**E-S**-**-25-**-**1532**-**5.pd**-**f **-**-**-**1 Sc**-**reem**-**...62**-**741**-**.png**,-**� **-**PO**·**NE-0**-**-25**-**-117**-**89.p**-**df **----, ..-**  ■■!!!!P.S://[WWW.lllS agmm.com/](http://WWW.lllSagmm.com/)�  hn.P.s:lltwitter-com/DrGPJuck | | | | | | | |
| --- | --- | --- | --- | --- | --- | --- | --- |
| **G** | SuphasireeChantavarin  To:@g.c.pluck  PONE-D-25-11789.pdf  Thanks!  l've resubmitted the manuscript (file attached).  U.G1"'î.ë\fl�� -:fU'V17�U'VI{  Suphas.iree Chantavarin, Ph.D.  **Lecturer and Head of the Cognitive- Psychology Are-a**   - Faculty of Psychology, Chulalongkorn University Il Bangkok, Thailand | Q � | Reply | � | Reply all | *r'* | Forward e (;;)  Mon l/1 0/2025 9:44 AM |
