# Supplementary material for: A lexical decision task for rapid estimation of crystalized vocabulary knowledge in Thai
Source: PLoS One. 2026 May 4;21(5):e0348126. doi: 10.1371/journal.pone.0348126 (PMC13138683; doi:10.1371/journal.pone.0348126)
Supplement: Attachment — Submitted filename: Response to Reviewers.docx [file pone.0348126.s004.docx]

Response to Reviewers

| Reviewer 1 Comments | Action taken by authors |
| --- | --- |
| 1. Target Population and Terminology: The target population for the test is not clearly defined. Additionally, the manuscript inconsistently uses the terms “patients” and “participants,” which should be clarified. | In the penultimate paragraph of the Introduction we have added clarification of the target population, the new text on page 7, highlighted in Yellow reads: “The target population is adults who speak Thai as their main language”.  The word patient is now only used when describing people with neurological disorders undergoing neuropsychological assessment. Otherwise, people being assessed in research are described as participants. |
| 2. Conceptual Framing of Vocabulary Assessment: The definition of vocabulary assessment is drawn from literature on healthy adults, but many cited examples relate to verbal intelligence rather than vocabulary breadth. To align with the stated aim of measuring vocabulary knowledge, it would be beneficial to review work on lexical decision and yes/no vocabulary tests, particularly Meara’s research and LexTALE-inspired instruments. | New text has been added to the Introduction on pages 4-5, highlighted in blue. The new text reads: ‘‘One method, widely-used for language assessment in educational contexts, is to present a mixed array of words and pseudowords, and the test-taker must choose which they know, and which they do not. Such recognition yes/no tests have been widely-used for student placement within a language education context, because, despite their simplicity, achievement scores correlate very highly with scores on more comprehensive and lengthy language-skill assessments (Meara & Jones, 1988). They also appear to have favorable psychometric properties when compared to multiple-choice vocabulary assessment methods (Meara & Buxton, 1987). The success of those lexical-decision studies let to the development of LexTALE, a simple lexical-decision based method for assessing vocabulary acquisition in a range of second-language contexts (Lemhöfer & Broersma, 2012)’ |
| 3. Theoretical Framework: The distinction between word recognition and recall is relevant and well-covered in Laufer and Aviad-Levitsky’s work, which could further strengthen the discussion. A clearer theoretical justification is needed to explain how the test format captures vocabulary knowledge, especially given that forced-choice tasks may reflect perceived word-likeness rather than lexical knowledge per se. | New text has been added on page 6, the new text, highlighted in blue reads “This is underlined by second language acquisition studies that show that vocabulary recognition is generally easier than recall (Levitzky-Aviad & Laufer, 2013), and is a better predictor of reading comprehension because performance of the task requires a narrower range of cognitive processes, offering a purer performance measure (Laufer & Aviad–Levitzky, 2017).” |
| 4. Stimuli Transparency: Providing English translations for the Thai stimuli would enhance accessibility for readers unfamiliar with the Thai language. | English translations are now shown in Table 2 on pages 15-16 and also in the Text, highlighted in green. |
| 5. Item Characteristics: The paper should report the proportion and characteristics of low- vs. high-frequency words. This information is crucial to understand the lexical difficulty of the test. | The item characteristics of the 42 final items has now been added, highlighted in yellow on page 14: Twenty of these 42 words were relatively high-frequency words (defined as 1 word per million and above; *M* = 4.82, *SD* = 6.04, range = 1.01-23.3), and the average number of characters in this set was 3.35 (*SD* = 0.88, range = 2-5 characters). The remaining words were relatively low-frequency words (defined as lower than 1 word per million; *M* = 0.19, *SD* = 0.27, range = 0-0.99), and the average number of characters in this set was 4.64 (*SD* = 1.09, range 3-7 characters). |
| 6. Outcome Measures and Platform Limitations: The outcome measures for the lexical decision task are not clearly described. Additionally, Qualtrics may not be suitable for collecting accurate response times, and this limitation should be acknowledged. | The outcome measure is now explicitly described with new text on page 10, highlighted in Teal that reads ‘For task performance, the variable of interest is accuracy, that is, whether the correct word in each pair is selected or not.’  The limitation of response time data is now acknowledged in the Discussion on page 29. Highlighted in Teal, the new text reads: ‘An additional issue with data collection online using Qualtrics was the limited accuracy of response time data. However, the only use made of times was for excluding participants who completed the full task unusually quickly or slowly, thus no fine timing at the sub second level was required.’ |
| 7. Methodological Justification: Selection criteria, data cleaning procedures, and item analysis should be supported by relevant literature to enhance methodological rigor. | Methodological justification has been strengthened with new text and citations added, highlighted in magenta on page 8: ‘Although these were recruited through convenience sampling, this approach is useful as it allows recruitment from a set geographical area, and convenience samples can be appropriate when used to pilot assessments and generate material for later studies (Golzar et al., 2022), as is being done in the current study.’  And on page 11: ‘, a behavior associated with low data quality (Zhang & Conrad, 2014).’  And on page 11: ‘Using combined geographical filtering and IP address is an effective method for reducing fraudulent data produced by bots or multiple attempts by the same individual (Donkin et al., 2025).’  And on page 13: ‘This pruning was necessary as items with very low variance would not be psychometrically useful, in that low variance within items tends to reduce scale reliability (Tsang et al., 2017)’ |
| 8. Interpretation of Test Format: It remains unclear whether the task truly measures vocabulary knowledge. The format, which asks participants to choose between a real word and a nonword, may primarily assess word-likeness judgments rather than vocabulary breadth. This should be addressed in the theoretical rationale. | Text addressing that issue has been added to the theoretical rationale supplied in the Introduction. The new text, highlighted in yellow on page 6 reads ‘Nevertheless, current models of lexical decision task performance suggest that multiple sources of information are used to reach a decision, and this includes how ‘word-like’ the pseudowords seem (Perea et al., 2020). It is therefore important to minimize such information, by providing only convincing pseudowords.’ |
| 9. Study 2 – Criterion Measures: The purpose and nature of the criterion tasks in Study 2 are unclear. In particular, the description of “verbal fluency tasks” lacks detail, making it difficult to determine whether they assess spoken or written fluency. For example, the description on p.19, line 391, suggests a written task, which needs clarification. | Extra clarification is provided on page 21, new text highlighted in khaki reads ‘Although the verbal output of the participant is oral, the words are written down by the test administrator.’ |
| Useful references:  1. Vermeiren, H., Vandendaele, A., & Brysbaert, M. (2023). Validated tests for language research with university students whose native language is English: Tests of vocabulary, general knowledge, author recognition, and reading comprehension. Behavior research methods, 55(3), 1036–1068. https://doi.org/10.3758/s13428-022-01856-x  2. Lee, S. T., van Heuven, W. J. B., Price, J. M., & Leong, C. X. R. (2025). Assessing bilingual language proficiency with a yes/no vocabulary test: the role of form-meaning vocabulary knowledge. Bilingualism: Language and Cognition, 1–17. https://doi.org/10.1017/S1366728924001007  3. Aviad-Levitzky, T., Laufer, B., & Goldstein, Z. (2019). The New Computer Adaptive Test of Size and Strength (CATSS): Development and Validation. Language Assessment Quarterly, 16(3), 345–368. https://doi.org/10.1080/15434303.2019.1649409  4. Schmitt, N., Nation, P., & Kremmel, B. (2020). Moving the field of vocabulary assessment forward: The need for more rigorous test development and validation. Language Teaching, 53(1), 109–120. https://doi.org/10.1017/S0261444819000326 | Several of these suggested references have been used (highlighted in magenta in the text), as well as several by the authors suggested in the previous comments. |

| Reviewer 2 Comments | Action taken by authors |
| --- | --- |
| 1. Introduction: The authors present the lexical decision task as a tool to identify the real word among a pair or group of letter strings and note the lack of standardised versions for clinical or research use. While this may apply to psychological research, such lexical decision tasks are also used in language sciences—for instance, the Dialang and LexTALE tests. The French LexTALE is even mentioned in the article, but not the English one. However, the task is different (yes/no recognition task). Some second language acquisition scholars suggest that these tasks are good proxies for general language competence. I suggest expanding the literature review to reflect this broader context or explaining why it does not need to be included. | New text has been added discussing LexTALE. That new text is highlighted in blue on page 4-5, and reads ‘One method, widely-used for language assessment in educational contexts, is to present a mixed array of words and pseudowords, and the test-taker must choice which they know, and which they do not. Such recognition yes/no tests have been widely-used for student placement within a language education context, because, despite their simplicity, achievement scores correlate very highly with scores on more comprehensive and lengthy language-skill assessments (Meara & Jones, 1988). They also appear to have favorable psychometric properties when compared to multiple-choice vocabulary assessment methods (Meara & Buxton, 1987). The success of those lexical-decision studies let to the development of LexTALE, a simple lexical-decision based method for assessing vocabulary acquisition in a range of second-language contexts (Lemhöfer & Broersma, 2012)’ |
| 2. Empirical Studies:  I recommend summarizing key participant characteristics in (a) table(s) for clarity. In the results section, including plots/graphs is necessary to help readers better visualize data distribution and relationships. | A table is provided summarizing participant characteristics. That is in a new table, Table 1 on page 12.  The plots are described below as they are suggested specifically in the annotated Word document provided by the reviewer. |
| Some clarifications are requested directly in the annotated version of the article. More detailed feedback is provided in this attached Word document. |  |
| The reviewer points below are cut and pasted from the Word document. |  |
| Abstract: [refereeing to correlations] ‘The term 'large' is not clear here. It would be helpful to specify the actual range or provide quantitative values to clarify what is meant.’ | Text has been added defining large within the abstract on page 2. Highlighted in red. |
| Introduction: ‘It would be beneficial to mention all available versions of the LexTALE test, not only for French but also for other languages such as English and German,and steps by step more languages. while LexTALE is primarily designed for second language acquisition research, some studies have shown that at least certain versions (e.g., French and German) do not exhibit ceiling effects even with native speakers. Including this information (or precising why you do it only for French) would provide a more comprehensive and nuanced view of the test's disponibilities. The Dialang test also use a lexical decision task. | Text has been added to cover these points, the new text on page 5, highlighted in red reads: ‘In addition to the English version of LexTALE, there are versions available for several different languages including French (Brysbaert, 2013), German and Dutch (Lemhöfer & Broersma, 2012). Although designed for use in second-language assessment, these tests often do not have ceiling effects in first-language speakers, suggesting they may have use in other contexts. Lexical decision is also one component of the DIALANG system, also used to assess competence of teenagers and adults learning a range of second languages (Zhang & Thompson, 2004).’ |
| Introduction: ‘Not really similar as it is a yes/no test’ | That text has been removed anyway to deal with the point above. |
| Introduction: ‘A table summarizing the caracteristics would be usefull.’ | That has been done, a new table is included (as described in the Reviewer’s 1^st^ point). |
| Method: ‘989 or 994?’ and a later comment ‘The number 994 appears later. What is correct?’ | The discrepancy is that the higher number is people who consented, and the lower number people who continued to answer questions after providing consent to participate. This is clarified in the text, new / altered text is highlighted on page 8 and 11 in yellow. |
| Method: [refereeing to the pseudoword creation] ‘How? By human or IA? | This is clarified in the text on page 9, highlighted in green, the new test reads ‘These were produced by a Thai doctoral level psycholinguist, without the use of AI.’ |
| Method: ‘So many participations in two days? Or was it two months?’ | It was two days. Many people shared the link, presumably they thought it a fun test. |
| Method: [Referring to exclusion of cases outside Thailand]. ‘Can you clarify why this step was necessary since you ask in the form about the languages of the participants (d)?’ | This was done to reduce the chance of invalid responses (e.g., bots) as the data was collected online. New text explaining that has been added on page 11, highlighted in magenta. |
| Results: [Referring to demographics] ‘A table summarizing the caracteristics would be usefull.’ | Table 1 on page 12 is now provided giving that summary. |
| Results: ‘Please also include this value in the number of participants, since percentages are not very intuitive with a sample size of less than 100.’ | The text has been added, highlighted on page 11 in yellow. |
| Results: [Referring to the correlation with gender] ‘Did you control for the effects of age and education to obtain this result, or could it simply be that the female participants were more highly educated or older?’ | This was a zero-order correlation, and that has been clarified in the text, highlighted on page 17 in red. We did not explore that any further with regression as it was not part of our research aim, and tangential directions with statistics could confuse the reader. |
| Results: “Could you please add the correlation plots? Eventually, could you add '(Study 1)' after 'online' and '(Study 2)' after 'interview' to make it clearer? | A new figure has been added, Figure 1. The captions and call out for that are highlighted in blue on page 18. Gender was not included in this as it is a point-biserial correlation and the scattergram is relatively uninformative. |
| Results: [Referring to the correlations of demographic variables] ‘Could you argue for using several correlations rather than one regression model? Have you checked for collider bias?’ | Bivariate correlations were used rather than a regression model, as we wished to identify and potential confounders and control for them later. This is now mentioned in the text on page 24, highlighted in blue ‘Bivariate correlations were performed to identify variables that may need to be controlled in later analyses.’ Collider bias would only be an issue if we attempted to control for multiple variables, by examining only bivariate correlations we have avoided any risk of collider bias. |
| Method (Study 2): ‘Add a table with important values regarding the participants to get the general image.’ | The new Table 1 on page 12 contains that information. |
| Method (Study 2): ‘Could you add a footnote about the proportion of Thai pupils who generally obtain a high school diploma? This varies greatly by country. Adding this information would help international readers to understand better.’ | That text has been added on page 20, highlighted in green. ‘The level of 40% of the sample with high school or less as their highest education level is similar to the national level, which is 50% (UNICEF, 2022).’ |
| Method (Study 2): ‘This paragraph may be worth putting under point 3.1.1, because as a reader, you will raise this question as soon as it is explained that both data collections happened at the same time.’ | The text mention has now been moved to that section (although the section numbering has been removed in this version), The section intended is named ‘Aims, study design, and hypotheses’ on page 19. |
| Results (Study 2): ‘Will these analyses be available in the online appendix?’ | A supplementary file is provided with that information. The call out for that is highlighted in green on page 33. |
| Results (Study 2): ‘This is confusing since they were run at the same time. Perhaps 'parallel online study' or 'online study (study 1)'? | Text has been added to clarify this, highlighted in blue on page 23, the next text read ‘the parallel online study (Study 1)’. |
| Results (Study 2) [Referring to Table 3, which is now Table 4]: ‘Could you please present it as a plot? | Plots have been provided for the overall performance measures of both fluency tasks, as well as the association with Boston Naming Test (requested by the reviewer in a later comment). The new figure is Figure 2, and the call out and caption are highlighted in red on page 25. |
| Results (Study 2): [referring to the Boston naming test] ‘Also as a plot’ | This is now provided as part of Figure 2 on page 25. |
| Results (Study 2): ‘The term 'high correlation' should be clearly defined before being used. I recommend specifying what range you consider to represent a 'high' correlation (as desvribed below), and then consistently applying that definition throughout the manuscript’. | The word ‘high’ has been changed to ‘large’ and new text has been added on page 17, highlighted in grey, the new text reads: ‘Qualitative descriptions of correlation coefficients used in this paper are based on a standardized interpretation of thresholds [34]: r > .1 = ‘small’, r > .2 = ‘typical’, and r > .3 = ‘large’).’ |
| Method (Study 3): ‘Could you please explain why it is 27? was the number of participants who were ready to continue, or was it based on some statistical modelling?’ | A description of this has been added, the new text on page 27, highlighted in yellow, reads ‘These were participants who indicated willingness to be assessed a second time, the sample size was based on that needed to detect r values > .5 at 80% power (Cohen, 1992).’ |
| Results (Study 3): ‘Add a plot please’ | A new figure has been added, figure 3. The callout and title are highlighted in green on page 28. |
| General Discussion: ‘Is this for L1 speakers? Or do you think it is also valid for L2 speakers, or should more items be included for them?’ | Text has been added to address this point, in the 1^st^ paragraph of the General Discussion, highlighted in dark green on page 29. The new text reads: ‘The task was designed for use in psychological research, and contains several quite low-frequency items. Although it could potentially be explored for use as a measure of Thai as a second language ability, it may be too difficult for less than advanced learners. Here we discuss in more detail some issues raised by the research.’ |

**Addition amendments**

Data repository information has been added.

The section numbering of the original submitted manuscript has been removed.

An error with the participants section of study 2 was corrected (the word ‘men’ was replaced with ‘women’).

An additional affiliation of the corresponding author and funding acknowledgements have been added.
